# Application of Polypyrrole Multi-Walled Carbon Nanotube Composite Layer for Detection of Mercury, Lead and Iron Ions Using Surface Plasmon Resonance Technique

**DOI:** 10.1371/journal.pone.0093962

**Published:** 2014-04-14

**Authors:** Amir Reza Sadrolhosseini, A. S. M. Noor, Afarin Bahrami, H. N. Lim, Zainal Abidin Talib, Mohd. Adzir Mahdi

**Affiliations:** 1 Wireless and Photonics Networks Research Center of Excellence (WiPNET), Faculty of Engineering Universiti Putra Malaysia, Serdang, Selangor, Malaysia; 2 Department of Computer and Communication Systems Engineering, Faculty of Engineering, Universiti Putra Malaysia, Serdang, Selangor, Malaysia; 3 Faculty of Science, Islamic Azad University, Eslamshahr Branch, Tehran, Iran; 4 Department of Chemistry, Faculty of Science, Universiti Putra Malaysia, Serdang, Selangor, Malaysia; 5 Functional Device Laboratory, Institute of Advanced Technology, Universiti Putra Malaysia, Serdang, Selangor, Malaysia; 6 Department of Physics, Faculty of Science, Universiti Putra Malaysia, Serdang, Selangor, Malaysia; US Naval Reseach Laboratory, United States of America

## Abstract

Polypyrrole multi-walled carbon nanotube composite layers were used to modify the gold layer to measure heavy metal ions using the surface plasmon resonance technique. The new sensor was fabricated to detect trace amounts of mercury (*Hg*), lead (*Pb*), and iron (*Fe*) ions. In the present research, the sensitivity of a polypyrrole multi-walled carbon nanotube composite layer and a polypyrrole layer were compared. The application of polypyrrole multi-walled carbon nanotubes enhanced the sensitivity and accuracy of the sensor for detecting ions in an aqueous solution due to the binding of mercury, lead, and iron ions to the sensing layer. The *Hg* ion bonded to the sensing layer more strongly than did the *Pb* and *Fe* ions. The limitation of the sensor was calculated to be about 0.1 ppm, which produced an angle shift in the region of 0.3° to 0.6°.

## Introduction

Polypyrrole, polypyrrole-chitosan, polyanilin, and polythiophene [1] are conducting polymers so they have a good environmental stability and the ability to monitor the intrinsic affinity to heavy metal ions [2], [3], [4,], glucose [5], [6], [7], H_2_O_2_ [5], [8], and they are sensitive to *Cu*, *Pb*, *Hg*, *Au* [9], [10], [11], *Zn* and *Ni* [12] ions. Hence, some researchers have considered the optical, electrical and thermal properties of conducting polymer.

Polypyrrole (PPy) is a well-known conducting polymer and used for biosensors [13], [14], composite materials [15], [16], microelectronic devices [17], while a combination of polypyrrole and nanoparticles was used to enhance the sensing of biomolecules [18].

Carbon nanotubes (CNTs) enhance the electrical, thermal and optical properties of polymer and plastic materials [19]. CNTs are cylindrical shells with diameters in the 100 nm range and a high surface to volume ratio. High sensitivity, fast reaction time, and the modulation behavior of CNTs near biomaterials are the prominent properties to apply when considering sensor applications [19], [20]. Hence, Sotiropoulou et al. [21] and Besteman et al. [22] sensed glucose using CNTs by immobilizing glucose-oxide-enzymes on CNTs. Moreover, CNTs were used to detect heavy metals [23] such as *Pb* [24], [25], *U* [26], and *Cd* [27] ions.

Mercury, lead and iron ions have long been recognized as toxic and harmful environmental pollutants. Mercury has high vapor pressure and low stability. The *Hg* element can release into the environment and produce extreme toxicity, the *Hg* ion combines primarily with inorganic compounds and cannot be methylated. In contrast, the mercuric ion combines with both inorganic and organic ligands and can be methylated [6]. Mercury [28] and lead have toxic effects on humans [29] and can cause severe damage in the bones, kidneys, liver, brain [30] and the central nervous system [31], [32], [33]. The iron ion is a heavy metal and cause environmental pollution, while an over load is a cause of hemochromatosis [34]. Therefore, the detection and measurement of *Hg*, *Pb*, and *Fe* ions become more significant. Hence, as layer detector, such as polypyrrole and 2-mercaptobenzothiazole [6], polypyrrole chitosan composite [7], 1,6-hexanedithiol [35], and apo-metallothionein [36] were used to detection heavy metal ions in an aqueous solution using surface plasmon resonance with different sensitivity and selectivity. Ning et al. also used Ag and Au nanoparticles for detection of *Hg* ion in a water sample [37]. Panta et al. combined the conventional electrochemical method, surface plasmon resonance (SPR), and magnetohydrodynamic (MHD) convection to measure the concentration of the *Hg* ion [38]. They detected the *Hg* ion down to 1 fM concentration in aqueous solution but this method was combination of three methods. Hence, the mentioned sensor layer are sensitive to *Hg* or *Pb* and they cannot detect the *Hg* and *Pb* together with a high degree of accuracy.

In 1994, Ajayan et al., reported synthesizing a carbon nanotube polymer nanocomposite [39]. Multi-walled carbon nanotubes (MWCNTs) have a conjugated π bond structure [40]. The delocalized π electrons in MWCNTs and PPy can bond together in a nanocomposite way to reduce the energy of the system and form a PPy/CNT nanocomposite [41], [42], [43], so polypyrrole with a MWCNT composite was used to improve the sensitivity and selectivity of sensors via interfacial interactions between MWCNTs and the conducting polymer [44, 45 and 46].

There are many techniques for analyzing trace metal, including atomic absorption, fluorescence spectrometry, inductively coupled plasma-mass spectrometry (ICP-MS), and electrochemical techniques. The application of these techniques suffers from disadvantages like the cost of the instrument, chemical knowledge, and nonlinearity of the calibration curve. A surface plasmon resonance (SPR) sensor is a versatile and effective optical technique to use to measure the concentration of ions and biomolecules. Good sensitivity, stability, reproducibility and portability are advantages of the SPR sensor. SPR is sensitive to a refractive index of analyte, and it exhibits change in real and imaginary parts of the refractive index [6], [47].

In the present work, PPy-MWCNTs films were characterized using the Surface Plasmon Resonance (SPR) technique. As mentioned above, mercury, lead and iron are the most important heavy metals, and detection of them are the subject of certain environmental research, so the detection of *Hg*, *Pb* and *Fe* ions are presented here using PPy-MWCNTs composite film.

## Materials and Methods

### Ions Solution

For preparation of the *Hg^2+^* aqueous solution, of *HgSO_4_* was dissolved in 1 liter of distilled deionized water (DDW) water producing in 1000 ppm of *HgSO_4_* solution. Then, other concentrations (0.1 ppm, 0.5 ppm, 1 ppm, 5 ppm, 15 ppm, 25 ppm, 50 ppm and 100 ppm) were prepared by a systematic dilution of the 1000 ppm of *HgSO_4_* solution. This process was repeated for the preparation of *Pb* and *Fe* ions in aqueous solution.

### PPy-MWCNTs Sensing Layer

The gold layer was prepared with a sputtering coating on a glass slide at a thickness of 49 nm prior to its use for electrochemical deposition of the sensing layer.

Multi-walled carbon nanotubes (Nanostructure & Amorphous Materials) and sodium dodecylbenzensulfonate (Sigma-Aldrich) were an analytical grade and thus used without further purification. The outside and inside diameters of MWCNT were 8 to 15 nm and 3 to 5 nm, respectively. The length and purity of the carbon nanotubes were 10 to 50 µm and >95%, respectively.

The PPy-MWCNTs layer were synthesized by electrochemical polymerization of distillated pyrrole on MWCNT. After dissolving the sodium dodecylbenzensulfonate (SDBS) in distilled water, the MWCNTs, with different weight ratio (0.3, 0.5, 0.7, 0.9, 1.1%) to the pyrrole monomer, were dispersed in a SDBS solution. The suspension sonicated for four hours to enhance the disaggregation of any nanotube bundles. The ratio of nanotubes to SDBS was 1∶10. Then the, pyrrole was dissolved in the MWCNT/SDBS solution and stirred. The PPy-MWCNT premixed solution was electropolymerized at +0.7 V for 5 sec in a three electrode electrochemical cell in which gold coated glass was used as a working electrode. A graphite rod and a saturated calomel electrode were used as the counter and reference electrode, respectively. The glass substrates were deposited with a thin, semi-transparent gold layer.

The electrochemical polymerization was performed using an electrochemical instrument (Autolab PGSTAT 101) at room temperature. The final PPy-MWCNT thin films were washed with water and methanol to remove the electrolyte solution and dried under a vacuum at room temperature for 24 h.

### PPy Sensing Layer

The PPy sensing layer was deposited on a gold coated high index glass slide using electrochemical deposition as reported in [48], [49]. Briefly, the electrochemical deposition of PPy was carried out using a potentiostat (Autolab PGSTAT 101). The polymers were potentiostatically prepared in a solution containing 0.3 M Pyrrole (pre-distilled), 0.1 M p-toluene sulfonate (P-TS) dopant at room temperature.

### SPR Setup and Experiment

**Figure 1:**
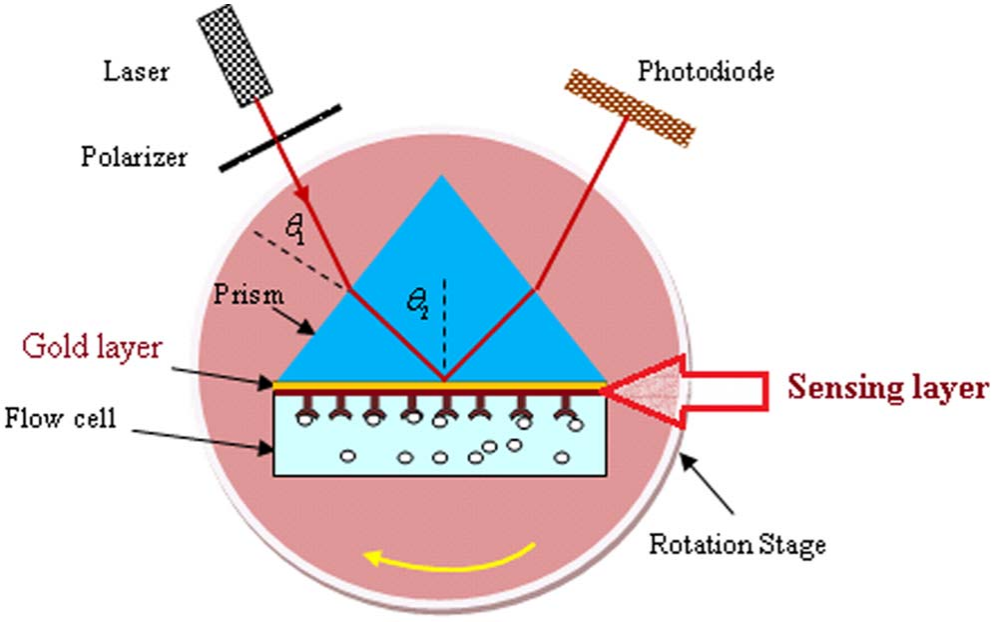
The SPR setup contains a He-Ne laser, a polarizer, a photodiode, a flow cell, a rotation stage, and a high refractive index prism.

The SPR setup was based on the Kretschmann configuration [50], [51] and shown in Figure 1. The transverse mode (TM mode) of the electromagnetic wave was achieved via the polarizer and delivered through a prism (SF52, Foctek). The PPy-MWCNTs layer was prepared on a gold coated glass slide (high index class SR52), attached to the high refractive index prism using an index-matching liquid (F-IMF-105, Newport, USA). The prism and flow cell, which contained the liquid sample, were placed on a rotation stage and rotated up to 30° at increments of a 0.016° step size. The TM mode for the He-Ne laser was excited to surface plasmon waves at the interface of the sensing layer and the sample. When the rotation stage was momentarily stopped, the variation of light intensity detected by the photodiode and the SPR signal were both registered. The experiment was repeated more than 10 times for each sample at room temperature.

The sensing layers with different percentages (0.3%, 0.5%, 0.7%, 0.9% and 1.1%) of MWCNTs were attached separately to the prism. When the distilled deionized water (DDW) passed through the flow cell, the SPR signals were registered to obtain the real and imaginary parts of the PPy-MWCNTs sensing layers. In addition, the distilled water was initially passed through the flow cell to determine a base line for sensing the *Hg, Pb*, and *Fe* ions using PPy-MWCNTs with 0.7% MWCNTs.

Kretschmann’s configuration is commonly used for the SPR method. In this current configuration, a gold layer was sandwiched between the prism and the sensing layer. The surface plasmon waves (SPW) propagated on the interface between the two materials, for example, metal (the gold layer) and dielectric (the sensing layer). The energy and momentum conservation must be satisfied to excite surface plasmons. Thus, the momentum of the incoming light must be equal to the momentum of the plasmons. The angle of resonance is a function of thickness and the optical properties of the gold layer and the sensing layer. The sensing layer covered the gold layer and adsorbed the ions, and the binding occurs. The accumulation of ions on the sensing layer could be detected by the SPR reflectivity signal because the refractive index and the thickness of sensing layer had changed. The thickness may change due to the binding of an ion to the surface of the sensing layer [52].

The resonance angle, reflectance, and the real and imaginary parts of the refractive index of sensing layers were calculated by minimizing the summation of experimental and theoretical reflectance,
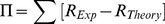
(1)Where 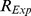 and 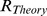 are the experimental and theoretical reflectivity, respectively; the theoretical reflectivity was achieved from the reflection coefficient (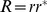, where 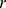 is the reflection coefficient.) [52], [53], [54]. In the present, the matrix method, which is explained in reference [51] was used [55] to analyze the data using Matlab software. The reflectivity is the function of refractive index and the thickness of gold layer and the sensing layer. Hence, the refractive index and the thickness of the sensing layer were found with the optimization and minimizing of Eq. (1). The detail of the calculation is presented in ref. [49], [51]. Further, the resonance angle was obtained after minimizing Eq. (1).

### Atomic Force Microscopy (AFM)

The sensing layer was tested before and after the adsorption *of Hg^2+^.* An AFM image at 600 dpi was acquired at a scan speed of 2 Hz with AFM (Q-scope 250, Agoura Hills) in tapering mode. The experiment was carried out at room temperature in ambient conditions.

### Inductively Coupled Plasma Mass Spectrometry (ICP-MS)

The concentrations of ions samples were also measured by inductively coupled plasma mass spectrometry (ICP-MS; Optima 2000 DV; Perkin Eimer), which was used to calibrate and test the measurement.

## Results and Discussion

### Optical Properties of PPy-MWCNTs Sensing Layer

**Figure 2:**
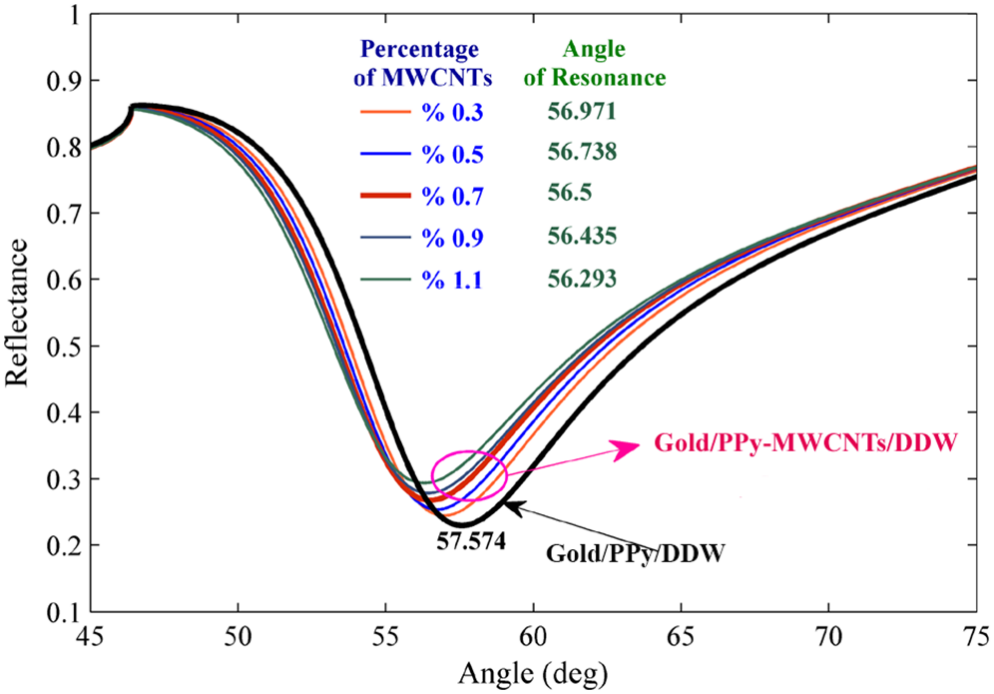
The SPR signals for different percentages of MWCNTs are used to measure the real and imaginary parts of a refractive index. The SPR signal for gold/PPy-MWCNTs/DDW and gold/PPy/DDW are the baseline signals used to obtain the angle shift after using the sensor for the detection of ions by minimizing the Eq. (1).

**Figure 3:**
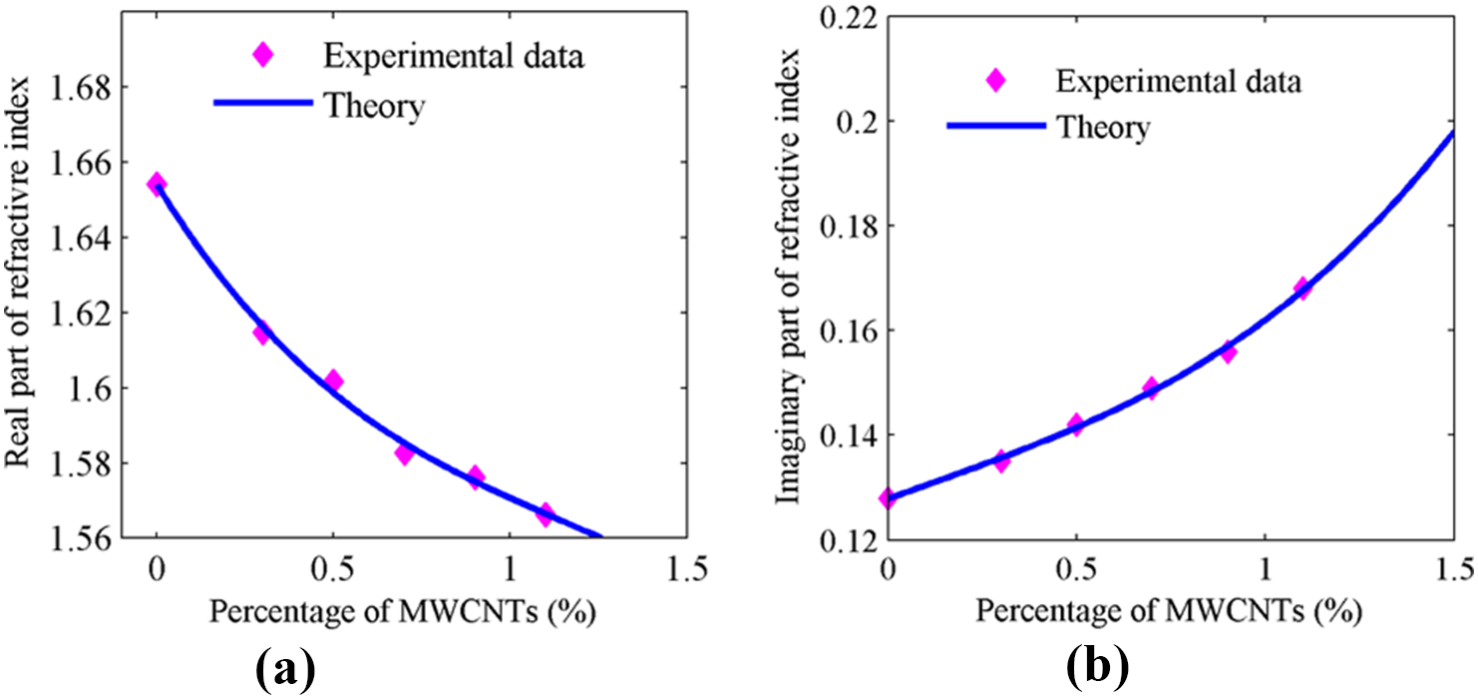
Variation of refractive index of PPy-MWCNTs for different concentration and the same thickness; a) real part of the refractive index; b) imaginary part of the refractive index.

The SPR signals for PPy-MWCNTs layers were obtained for different percentages of MWCNTs from 0.3% to 1.1% (Figure 2). The resonance angle decreased from 56.971° to 56.293° and reflectance shifted from 0.244 to 0.294 with the increasing percentage of MWCNTs. Figures 3(a) and 3(b) show the variation of real and imaginary parts of the refractive index versus the percentage of MWCNTs. The refractive index of the layer was obtained using Eq. (1) [51]. As shown in the experimental results, the imaginary part increased with an increase in the percentage of MWCNTs because the conductivity of the layers increased [56]. Moreover, the real part of the refractive index monotonically decreased as expected from the Kramers–Kroning relationship [47]. The optical parameters of the sensing layer are sorted in Table 1 at the same thickness of about 21 nm.

**Table 1:** Pertinent parameters of PPY-MWCNTs layer.

Percentage of MWCNTs	Real part of refractive index	Imaginary part of refractive index
**0.3**	1.6148	0.135
**0.5**	1.6017	0.142
**0.7**	1.5826	0.149
**0.9**	1.5762	0.156
**1.1**	1.5663	0.168

### Detection of Ions

In this work, the SPR sensor was based on angular modulation, and the variation of the resonance angle was considered for detection of heavy metals. Hence, the baseline was achieved prior to carrying out the experiment. Figure 2 shows the SPR signals of the gold layer/PPy/DDW and the gold layer/PPy-MWCNTs/DDW for baseline the sensor, and the percentage of MWCNTs was % 0.7. Hence, the angle of resonance for baselines at the first and second (MWCNTs % 0.7) configuration were 57.574° and 56.5°, respectively.

**Figure 4:**
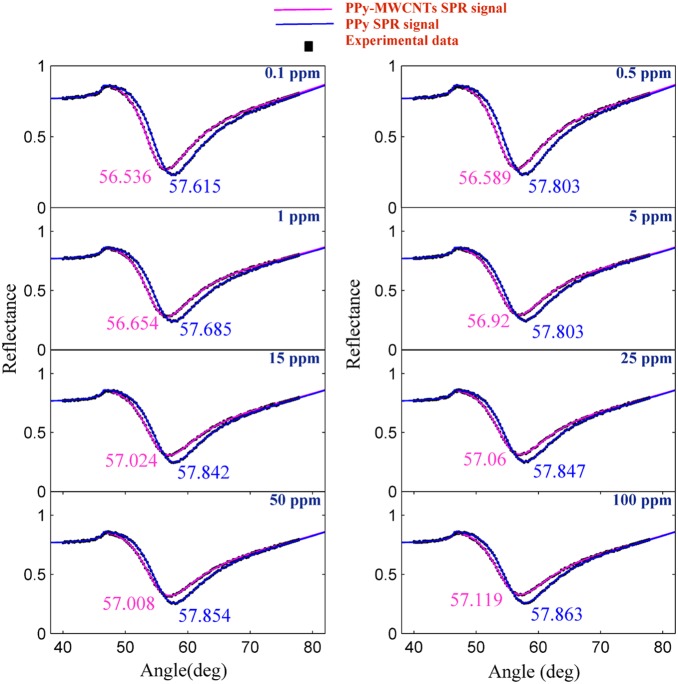
SPR signals to detect the *Hg* ion in an aqueous solution at the saturated values.

**Figure 5:**
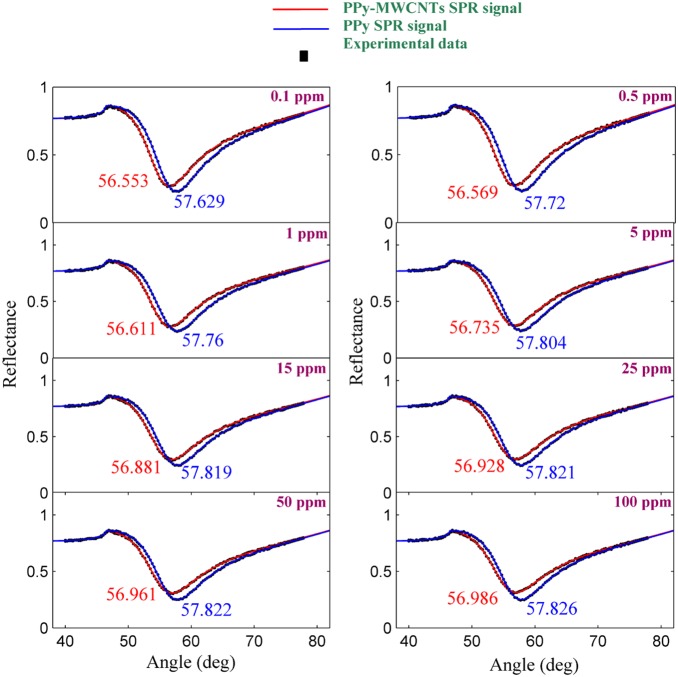
SPR signals for a determination of *Pb* ion concentration in an aqueous solution at the saturated values.

**Figure 6:**
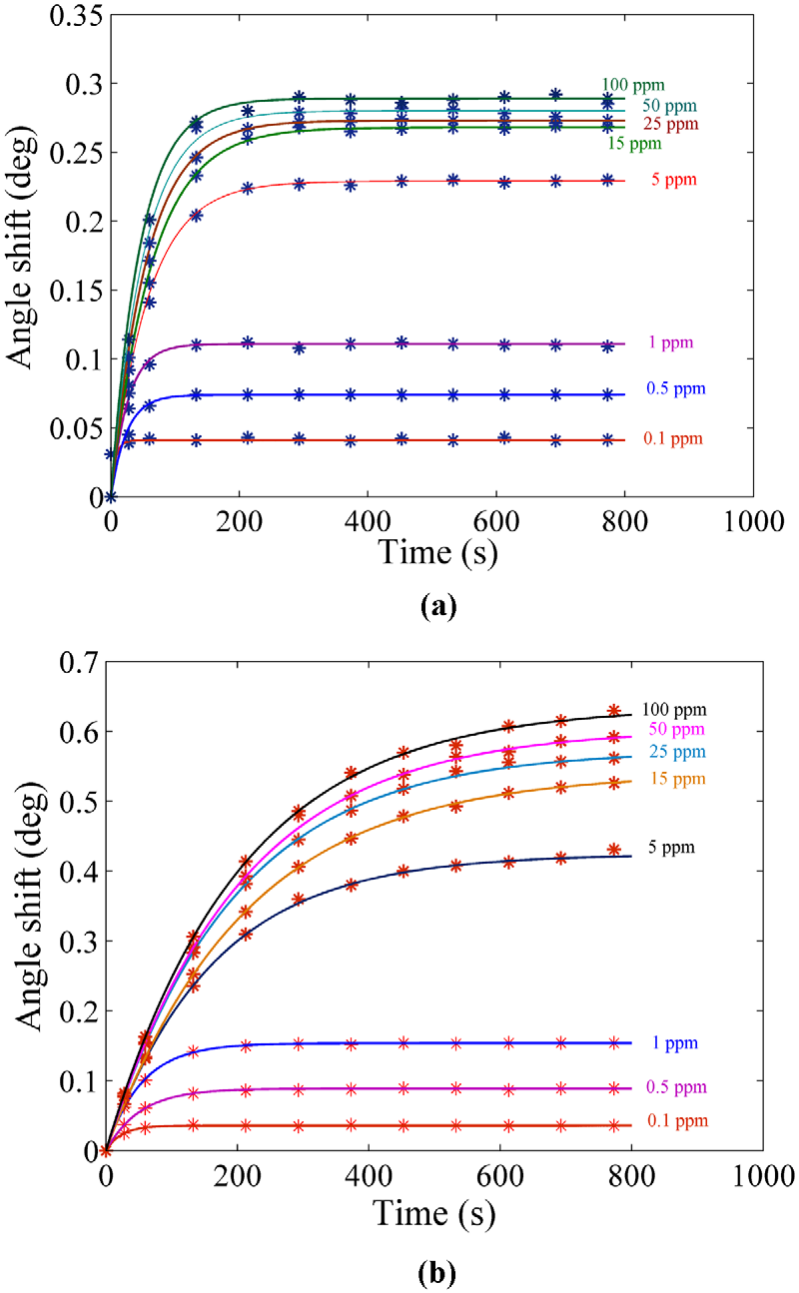
Sensogram for each concentration of the *Hg* ions: a) PPy sensing layer b) PPy-MWCNTs.

**Figure 7:**
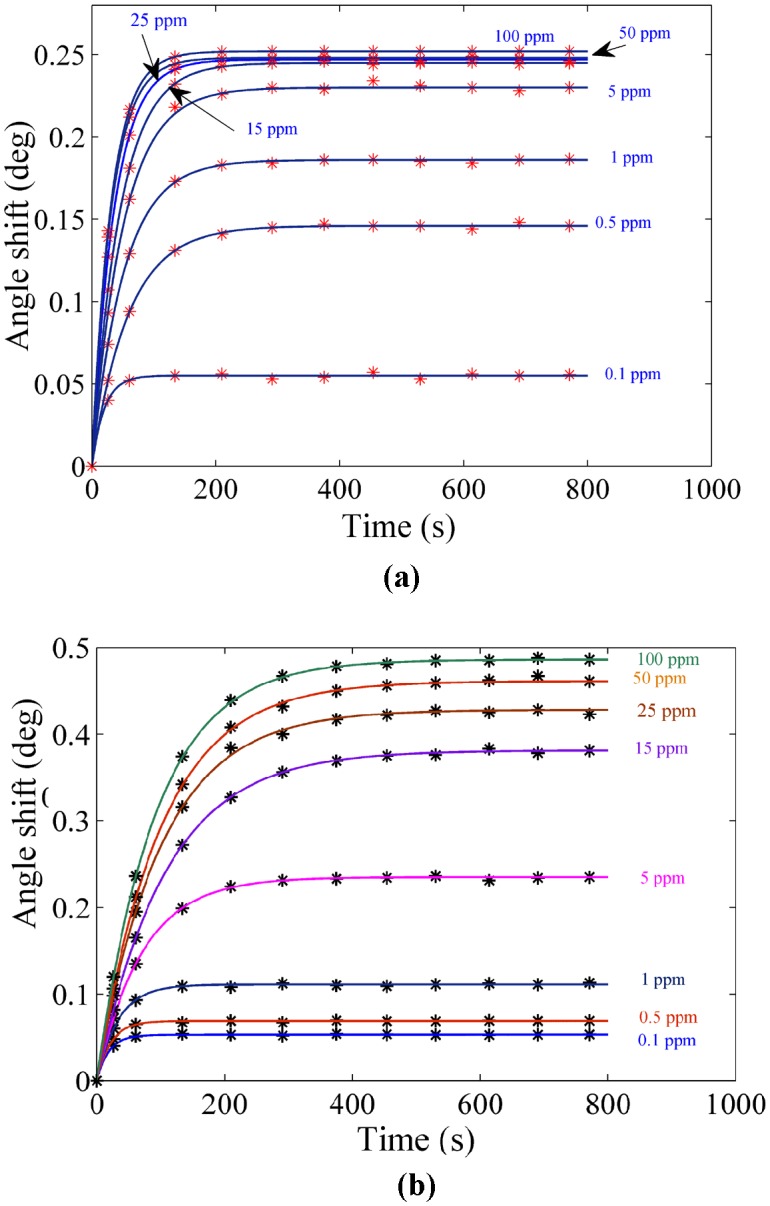
Sensogram for detection of the *Pb* ion in different concentration: a) PPy sensing layer b) PPy-MWCNTs sensing layer.

**Figure 8:**
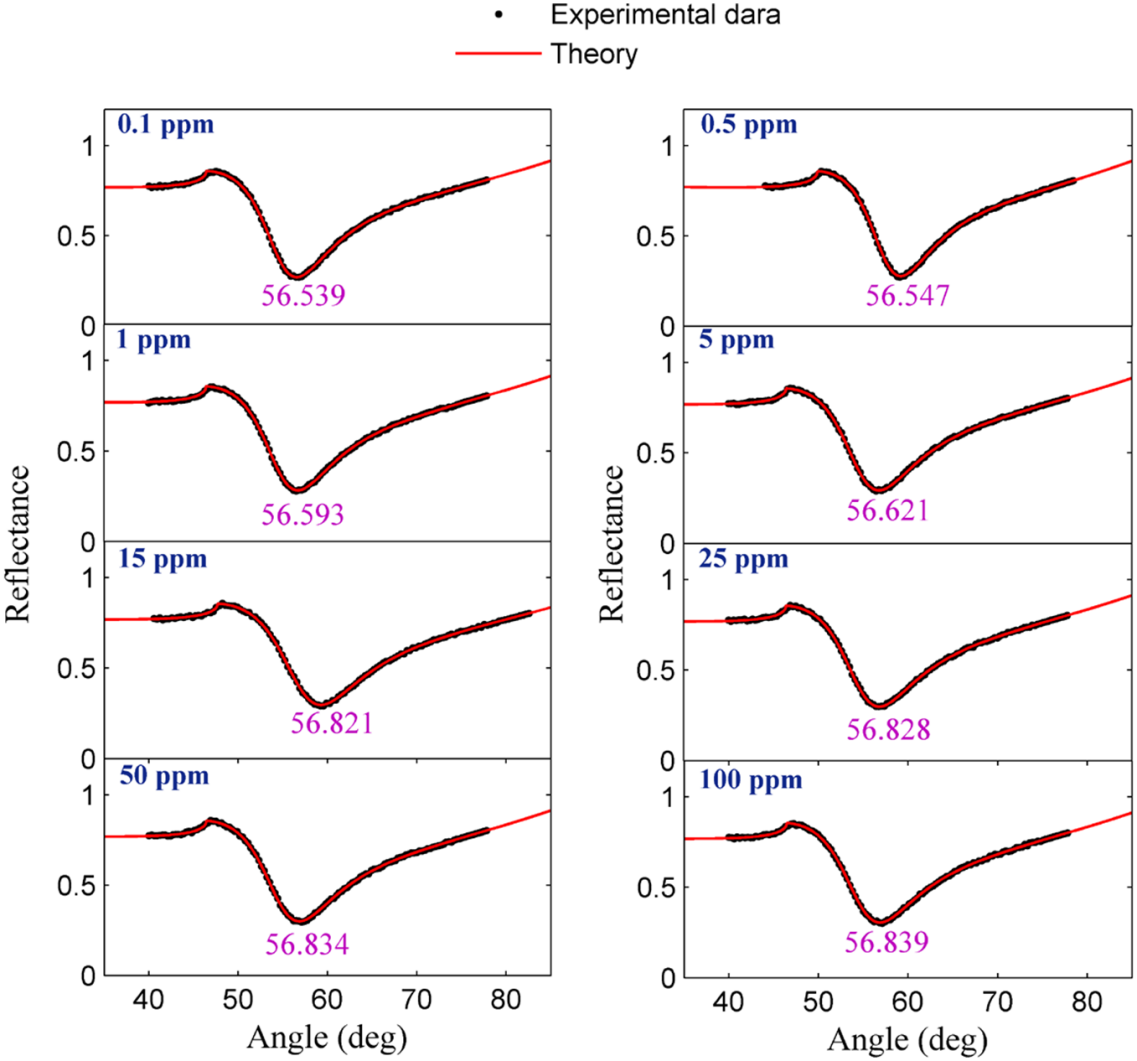
SPR signals related to determining the concentration of *Fe* ion in an aqueous solution at the saturation value.

**Figure 9:**
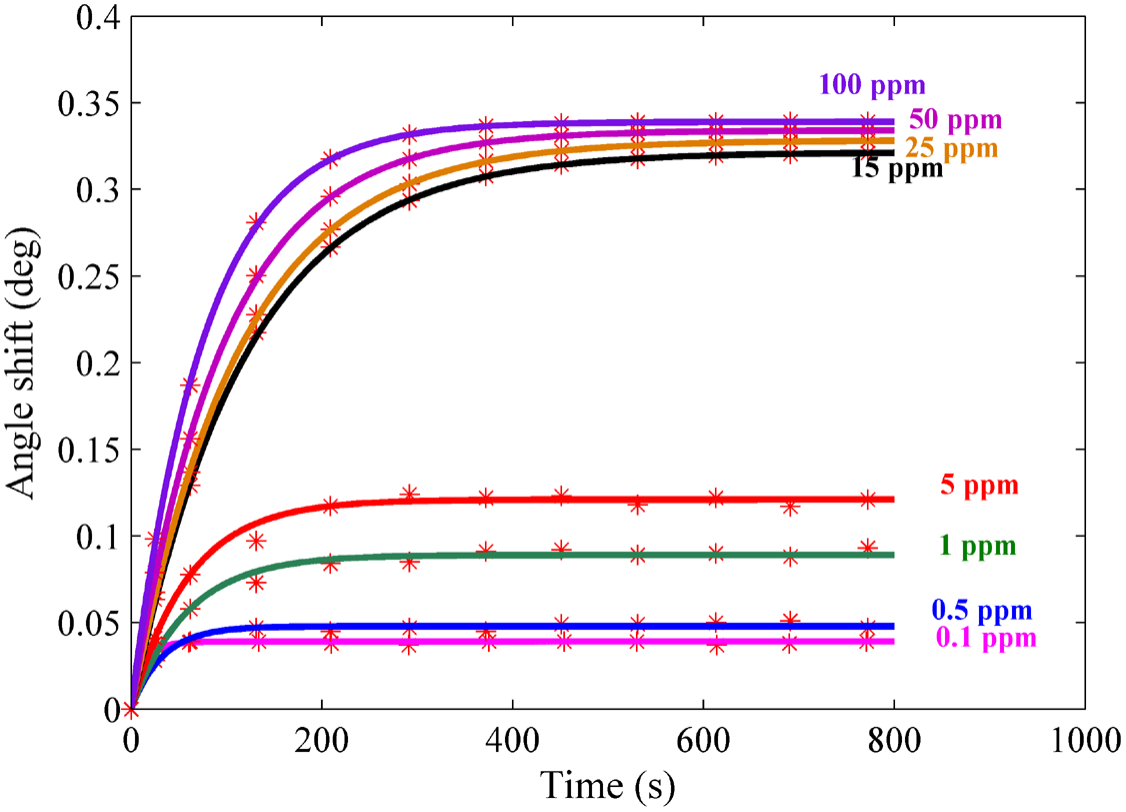
Sensogram for different concentration of the *Fe* ion detected using PPy-MWCNTs.

The experiment was carried out for each sample separately. The solution containing the ion was loaded in the flow cell, and the experiment was repeated for 780 s to register the SPR signal. Figures 4 and 5 show the SPR signal at saturated value for *Hg* and *Pb* ions, and typical sensograms for different concentration of *Hg* and *Pb* ions are depicted in Figures 6(a), 6(b), 7(a) and 7(b) that were derived from SPR signals during the experiment with PPy and PPy-MWCNTs sensing layers, respectively. Figures 8 and 9 show the SPR signals at saturation value and sensograms for different concentration of *Fe* ion that were detected using PPy-MWCNTs sensing layer, respectively. Moreover, these results were compared with the results (SPR signals and sensograms) for detection of *Fe* ion using the PPy sensing layer which were presented in ref [49], and the numerical data was sorted in Table 2. In figures 4, 5 and 8, the solid square (▪) and solid line are the experimental data (*R_Exp_*) and the theoretical reflectivity (*R_Theory_*) [54], [57], [58], respectively. The theoretical reflectivity was fitted to experimental data with correlation coefficients greater than 0.96 for each sample.

**Table 2:** Pertinent parameters of PPy and PPy-MWCNTs sensing layer were derived from Langmuire’s first order adsorption model.

PPy/MWCNTs *K* Δ*θ_max_*			PPy *K* Δ*θ_max_*	
***Hg***	0.3573	0.6273	0.6881	0.2912
***Pb***	0.206	0.5064	2.781	0.2501
***Fe*** a	1.225	0.3387	0.5186	0.2028

a The experimental data for detection of *Fe* ion using the PPy was achieved in ref [49].

The resonance angles increased related to the baseline with the time passage before the saturation value. In the results, the angle shift and the saturation value of PPy-MWCNTs in the sensing layer were higher than the PPy sensing layer for the same concentration. The saturation value and the rate constant of the sensing layers for detecting the *Hg, Pb* and *Fe* ions were obtained via fitting Langmuire’s first order adsorption model to the experimental data as follows [53]:
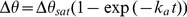
(2)Where 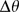, 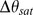, 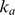 and 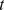 are the angle shift, the saturated value of the resonance angle shift, the rate constant, and the response time of the sensor, respectively.

**Figure 10:**
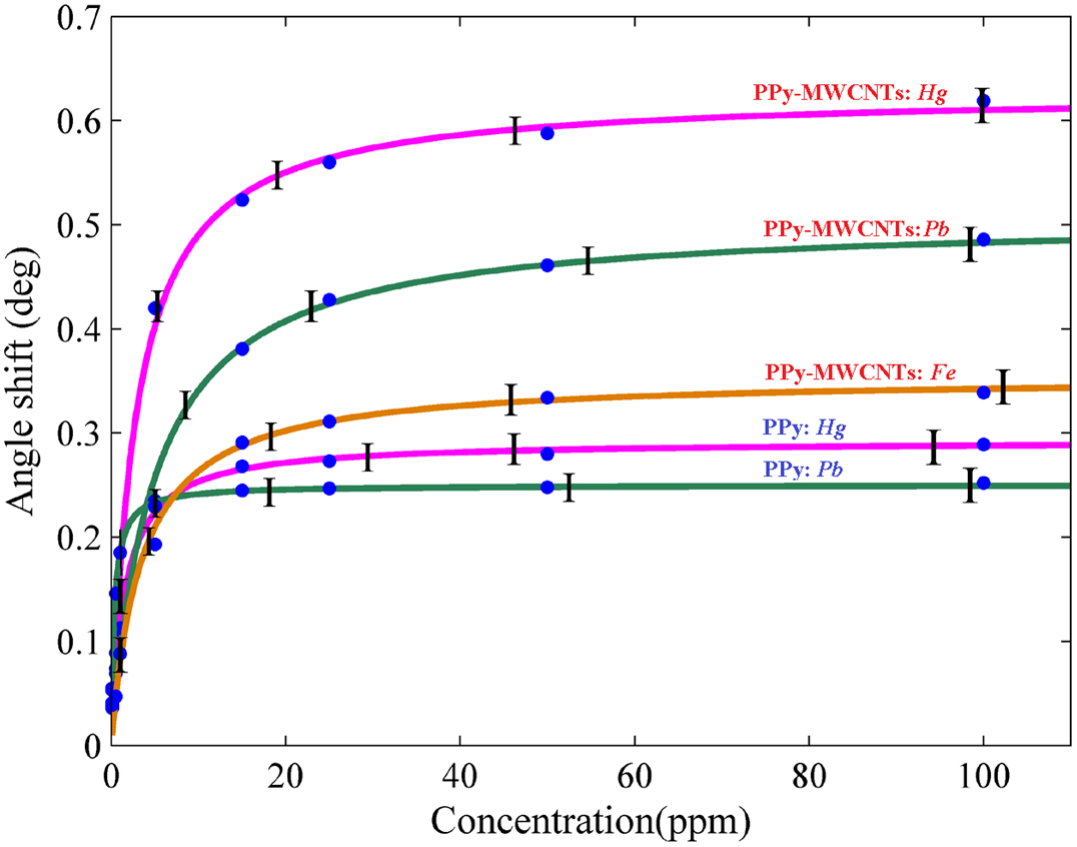
Angle shift at saturation value versus the concentration of ions for determination of the ion value in aqueous solution.

Figure 10 shows the variation of angle shift versus the concentration of ions. These data were derived from the sensogram diagram at a saturation value. The experimental data had a good fit to the Langmuir equation [59].
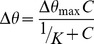
(3)where 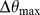, 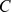 and 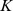 are the maximum value of resonance angle shift, the concentration of ions, and affinity constant, respectively. The parameters are displayed in Table 2.

The increase of the SPR angle for the PPy-MWCNTs layer when compared to the PPy sensing layer indicated that MWCNTs incorporated inside the PPy structure. The sensograms shown in Figures 6, 7 and 9, monitored the binding that accrued for the *Hg, Pb*, and *Fe* ions in the PPy-MWCNTs layer. The composite films for PPy-MWCNTs indicated the higher angle shift compared to the PPy sensing layer for each time at the same concentration. Indeed, the carbon nanotube improved the interaction of the sensing layer and enhanced the sensitivity of the sensor.

**Figure 11:**
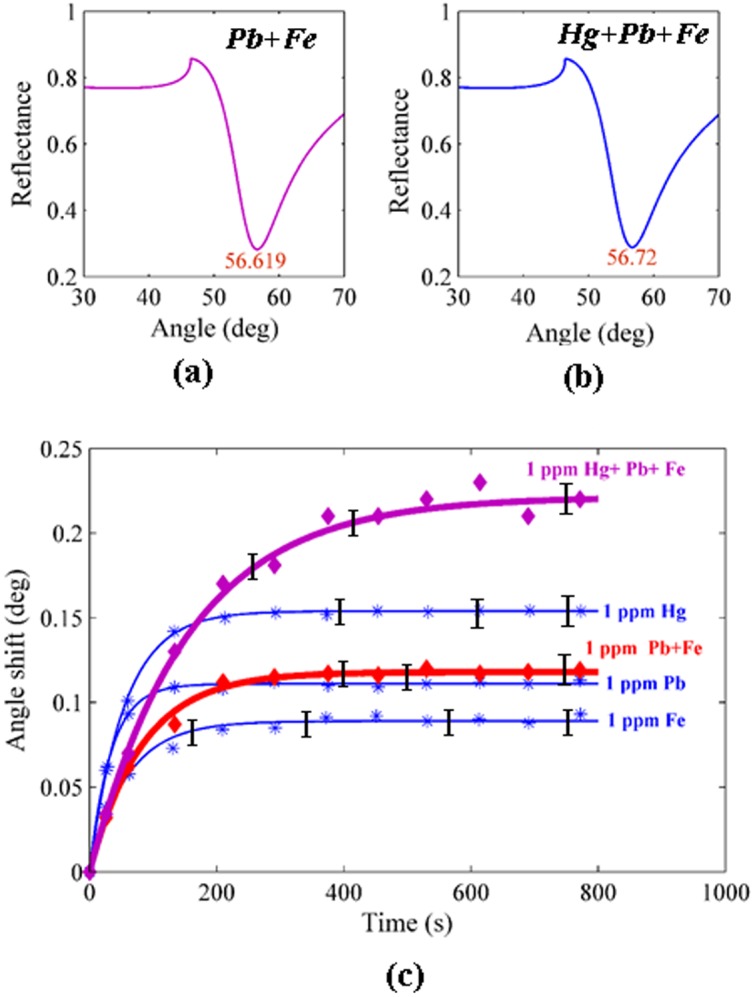
SPR signal and sensogram related to mixture of a) *Pb* and *Fe* ions at saturation value; b) *Hg, Pb* and *Fe* ions at saturation value c) Sensogram for detection of ions in the mixture.

To show the sensitivity of the PPy and PPy-MWCNTs sensing layer for the *Hg, Pb* and *Fe* ions, the calibration curves shown in Figure 10 were drawn for the sensors. These plots show the enhancement of sensitivity for detecting ions in the presence of MWCNTs explicitly, and also that the variation of the angle shift (Δθ_max_) for the *Hg* ion was higher than for the other ions. The experiment was repeated 5 times for each concentration, and in accordance with the standard deviation of angle shift were in the region of 0.0045° to 0.057°, the error bars were obtained. In addition, Figure 11 (a), 11(b) and 11(c) show the SPR signals and the sensogram for the mixing of ions, respectively. The sensogram for a mixture of *Hg*, *Pb* and *Fe* was higher than sensogram for mixing of *Pb* and *Fe*. Hence, the sensor could detect the *Hg* ion better than the *Pb* and *Fe* ions (*Hg*>*Pb*>*Fe*). The concentration of *Hg*, *Pb* and *Fe* ions in the mixture were 1.03 pm, 1.02 ppm and 0.99 ppm, respectively. After the aqueous solution contacted the surface of the sensing layer, the binding occurred between the ions and the sensing layer, and the sensing layer adsorbed the ions. The concentration of the remainder ions in the aqueous solution was measured after the experiments using ICP-MS. In addition, the sensing layers that used in experiment was immersed in HNO_3_ (2 mol) and ions (*Hg* or *Pb* or *Fe*) released from sensing layer. The concentration of released ions was measured using ICP-MS with limitation of 0.01 mg/L. These results are sorted in Table 3.

**Table 3:** The concentration of ions in the mixture and attached the sensing layer were measured using ICP-MS before and after the experiment.

	*Hg* (mg/L)	*Pb* (mg/L)	*Fe* (mg/L)
**Concentration of ions in the mixture before the experiment**	1.03	1.02	0.99
**Concentration of ions in the mixture after the experiment**	0.06	0.11	0.2
**Concentration of ions attached on sensing layer after the experiment**	0.96	0.84	0.71

**Figure 12:**
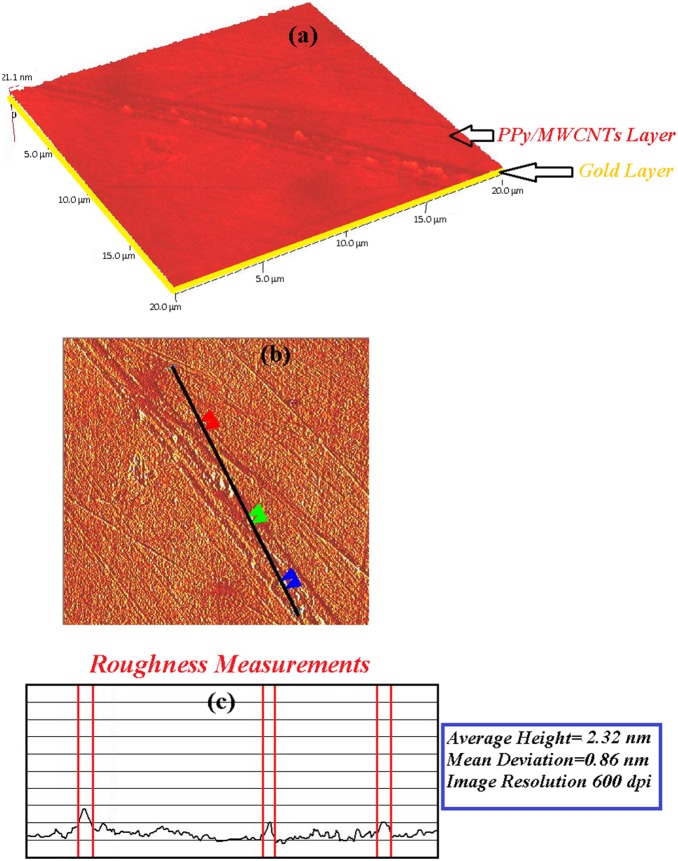
The AFM image for PPy-MWCNTs before the adsorption of the *Hg* ion. The roughness of layer was about 2.32 nm.

**Figure 13:**
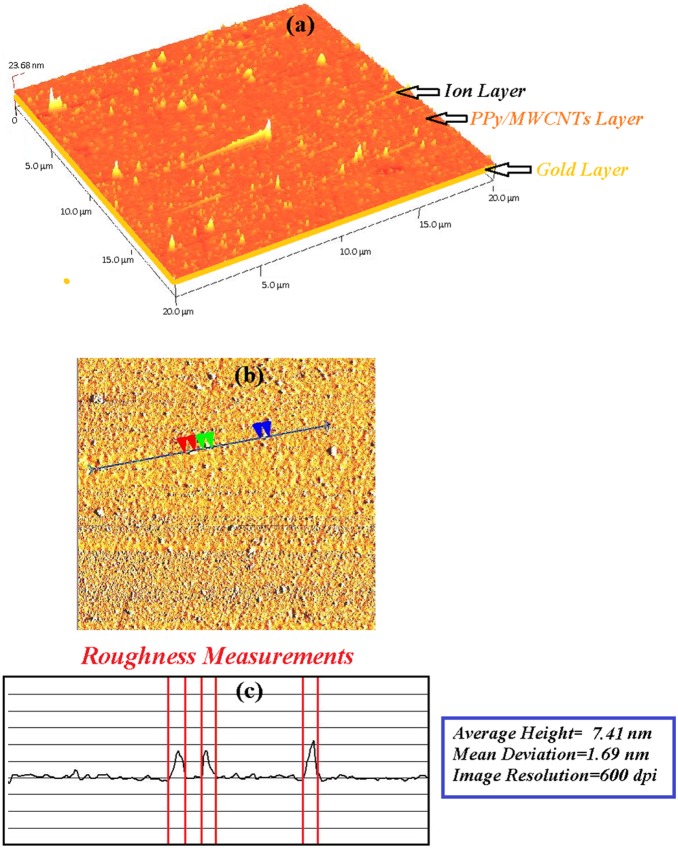
The AFM image after the sensor layer adsorbed the *Hg* ion. The roughness of the layer increased to 7.41 nm.

The sensing layer (PPy-MWCNTs) was deposited and covered the surface of the gold layer. As shown in Figures. 12 and 13, the morphology of the sensing layer changed after contact with the *Hg* ion (5 ppm). Undoubtedly, the layer adsorbed the ion, and the thickness and roughness of the layer increased from 21.1 nm to 23.68 nm and 2.32 to 7.41 nm, respectively. Hence, the SPR angle shift increased with the binding of the ion to the surface of the PPy-MWCNTs layer.

PPy-MWCNTs is a stable layer and some solutions, such as oil and fuel [51] cannot dissolve it. Moreover, the variation of resonance angle shift for the detection of *Hg* ion in the presence of PPy-MWCNTs is larger than the resonance angle shift that is reported in the literature, and the sensitivity of PPy-MWCNTs is higher than PPy [6] and PPy-CHI [7].

## Conclusion

The PPy-MWCNTs sensing layer was successfully used to detect low concentrations of *Hg*, *Pb*, and *Fe* ions in aqueous solutions. The MWCNTs improved the sensitivity of the SPR sensor, such that the angle shift increased relative to the angle shift of the PPy sensing layer to measure the concentration of mentioned ions. The calibration curve and the concentration of ions (ICP-MS results) which were binding to sensing layer showed the PPy-MWCNTs sensing layer is convenient for monitoring the low concentrations of ions. The limitation of sensor is about 0.1 ppm and can select the *Hg* ion better than the *Pb* or the *Fe* ions. Although, the accuracy of ICP-MS is higher than SPR sensor with PPy-MWCNTs but the SPR sensor depends on the refractive index of sample, and it is simpler, cheaper and more compact than ICP-MS and other spectroscopy methods.
